# A Systematic Review and Meta-analysis on the Prevalence of Low Birth Weight Infants in Iran

**DOI:** 10.1155/2020/3686471

**Published:** 2020-10-15

**Authors:** Maryam Sabbaghchi, Rostam Jalali, Masoud Mohammadi

**Affiliations:** ^1^Department of Demography, Faculty of Social Sciences, Yazd University, Yazd, Iran; ^2^Department of Nursing, School of Nursing and Midwifery, Kermanshah University of Medical Sciences, Kermanshah, Iran

## Abstract

**Background:**

Low birth weight is a significant index for survival, intrauterine growth, and mortality in infants. Thus, this study is aimed at determining the prevalence of low birth weight in Iranian infants through a systematic review and meta-analysis.

**Methods:**

This study was performed by meta-analysis from January 2000 to December 2019. The studies relevant to the topic have been obtained through search in databases of Scopus, ScienceDirect, SID, Magiran, Barakat Knowledge Network System, Medline (PubMed), and Google Scholar. Heterogeneity of the studies has been assessed by the *I*^2^ index, and data analysis was done using Comprehensive Meta-Analysis software.

**Results:**

By investigating 14 articles and 93924 infants, the total prevalence of low birth weight in infants in Iran was achieved at 8.5% (95% CI: 7.3-9.9%) according to the meta-analysis; the most prevalence of low birth weight was in infants in Hamedan at 19.1% (95% CI: 21.2-17.2%) in 2007, and the lowest prevalence of low birth weight was in infants in Tonekabon at 4.2% (95% CI: 3.4-5.2%) in 2005, and also, by increasing the sample size, the prevalence of low birth weight increases, by which the difference is statistically significant (*P* < 0.05).

**Conclusion:**

Due to the high prevalence of low birth weight in infants in Iran, health policy-makers must take effective attempts in order to reduce it in infants.

## 1. Background

Intrauterine growth restriction (IUGR) refers to a condition in which an unborn baby is smaller than it should be because it is not growing generally inside the womb. Delayed growth puts the baby at risk of specific health problems during pregnancy and delivery and after birth. They include low birth weight; difficulty handling the stresses of vaginal delivery; decreased oxygen levels; hypoglycemia; low resistance to infection; low Apgar scores; meconium aspiration, which can lead to breathing problems; trouble maintaining body temperature; and abnormally high red blood cell count [1–5].

A common cause is a problem with the placenta. The placenta is the tissue that joins the mother and fetus, carrying oxygen and nutrients to the baby and permitting the release of waste products from the baby. The condition can also occur as the result of specific health problems in the mother, such as advanced diabetes; high blood pressure or heart disease; infections such as rubella, cytomegalovirus, toxoplasmosis, and syphilis; kidney disease or lung disease; malnutrition or anemia; sickle cell anemia; smoking; drinking alcohol; or abusing drugs [2–6].

Low birth weight is the most important factor determining survival and an essential marker of intrauterine growth. It is also one of the most straightforward and most general health indices in each society and is a crucial index in infant mortality [1–3]. Approximately 16% of births lead to low birth weight infants annually, by which 70% of them result in death [4–6]. The World Health Organization named infants with a birth weight lower than 2500 grams as low birth weight (LBW) [7]; mortality in infants with weight lesser than 2500 g is higher 40 times, and mortality in infants with weight more minor than 1500 g is higher 200 times more than infants with weight greater than 2500 g [7].

In addition to spiritual and mental problems, the high costs of caring and treatments of LBW infants at birth are borne by their families, who often belonged to vulnerable society segments [8]; out of 25 million infants with LBW born annually, more than 90% of them are born in developing countries [9]; in the classification of causes for infants' mortality, low birth weight is the second important cause for infant death in 40-80% cases [6–9].

LBW infants are more susceptible to risks such as cerebral palsy, mental retardation, neural defects, pulmonary diseases, sudden death syndrome, and complications caused by hospitalization at intensive care units in comparison to healthy weight infants [10–12]. In the conducted studies in various regions in Iran, the prevalence of LBW was reported as 11.8% in Qom [13], 19.1% in Hamedan [14], 8.6% in Tehran [15], 7.7% in Babol [16], and 6.9% in Guilan [17], which is indicating inconsistency and ambiguities in the prevalence of LBW in infants in Iran.

Since low birth weight increases the rate of mortality, disability, and many diseases in childhood and adulthood, and since performing interventional studies to reduce the prevalence of low birth weight in infants require accurate and consistent information to prevent problems and complications of LBW, the question of this research is on the rate of the total prevalence of LBW in infants in Iran. This study is aimed at determining the incidence of LBW in infants in Iran through a systematic review and meta-analysis.

## 2. Methods

### 2.1. Search Strategy

This was a systematic review and meta-analysis done by extraction of results of conducted studies in the context of the prevalence of LBW in infants in Iran and consisted of articles published in domestic and foreign journals and literature review in databases of ScienceDirect, Scopus, SID, Magiran, Medline (PubMed), Barakat Knowledge Network System, and Google Scholar from Jan 2000 to Dec 2019. The literature review has been performed by using keywords of low-birth weight, infants, and Iran and also probable combinations in English and Persian language. Therefore, literature review in Persian-language databases has been done by using mentioned Persian-language keywords, and for literature review in English-language databases, the English equivalent words of low birth weight, birth, neonates, and Iran were used, and also, for searching in Google Scholar, every two words in Persian and English language have been used, and logical operators of AND and OR in combination have been applied in order to have complete access to all the articles. Therefore, the operator of OR has been applied to investigate common names in the cases such as (Low birth weight OR Low-Birth-Weight Infant OR Infant LBW) and (neonates OR Infant OR Newborn), and also, the operator of AND has been applied between keywords of (Low birth weight AND neonates) through matching the words in the MeSH browser.

### 2.2. Inclusion Criteria and Evaluation of Articles

Firstly, all the articles were gathered by using selected keywords, and after finishing the literature review, a list consisting of articles' abstracts was prepared.

After hiding the characteristics of articles, including the journal name and authors, the full texts have been provided to reviewers. Each article was studied by two independent reviewers. In case of rejection, the reason for refusal has been mentioned. In case of a lack of consensus between the two reviewers, the article was reviewed by a third reviewer, and the third reviewer's opinion was considered.

Articles in English and Persian language with cross-sectional design in the context of prevalence and frequency of LBW in Iranian infants were eligible to be enrolled in the study, and other review studies, case-control studies, cohort, and interventional studies were excluded from the article list. In this study, a review of gray literature, evidence, and documents not printed and published due to any reason was aimed at searching keywords in Google search and reviewing topic-relevant websites.

Then, investigated studies were evaluated based on the 4-stage process of PRISMA 2009 consisting of identification of articles, screening, assessing the criteria for accepting the articles, and finally assessing the included articles in the meta-analysis.

### 2.3. Qualitative Assessment

In order to assess the studies, the STROBE checklist was used. This checklist consisted of 22 sections, by which 18 items of it are general and are used for all observational studies including cohort, case-control, and cross-sectional studies, and 4 items of it are specified, which is related to the type of study, and include various aspects of methodology such as the study's objectives, appropriate determination of sample size, type of study, sampling method, study population, data gathering method, description of variables, the way samples were evaluated, data gathering tools, objectives assessed in the study, applied statistical test, and report of the results. Accordingly, the maximum score for qualitative assessment was considered 32, and articles with scores of lesser than 14 were considered poor based on assessment and evaluation and were excluded from the study.

### 2.4. Statistical Analysis

The prevalence of LBW in infants for each study conducted in Iran was achieved; heterogeneity of the studies has been investigated by the *I*^2^ test. Generally, heterogeneity has classified into three classes: heterogeneity less than 25% (low heterogeneity), between 25 and 75% (moderate heterogeneity), and more than 75% (high heterogeneity). Data were analyzed by Comprehensive Meta-Analysis software (Biostat, Englewood, NJ, USA, version 3). The probability of publication bias in the results has been evaluated by a Funnel plot and by using the Egger test at the significance level of 0.05, and also, to assess the impacts of active potential factors in the heterogeneity of studies, a metaregression test has been used in two elements of sample size and the year the study was performed in.

## 3. Results

**Figure 1:**
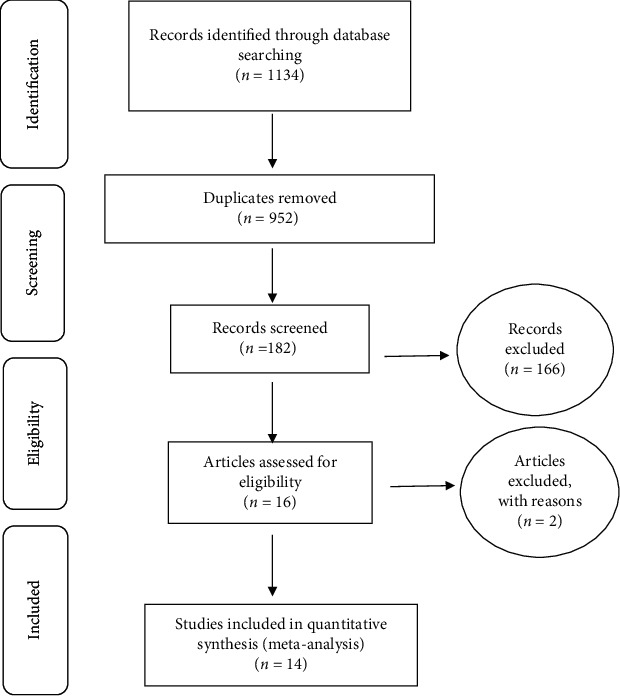
The flowchart on the stages of including the studies in the systematic review and meta-analysis (PRISMA 2009).

The results are based on conducted researches on the prevalence of LBW in Iranian infants, consisting of published articles in domestic and foreign journals. Literature review was done to 95 articles in databases of Magiran, SID, and Barakat Knowledge Network System, 185 articles in Medline (PubMed), 321 articles in ScienceDirect, 72 articles in Scopus, and 461 articles in Google Scholar, and 1134 articles were obtained based on the 4-stage process of PRISMA 2009 (Figure 1). Then, 182 eligible articles based on primary investigations after omitting 952 duplicate articles remained. Finally, 166 irrelevant articles have been omitted, and after secondary evaluation, two articles were also omitted due to lack of access to the abstract and full text and low quality of articles; 14 articles were finally entered into the meta-analysis process (Table 1) [13–26].

### 3.1. Evaluation of Heterogeneity and Publication Bias

**Figure 2:**
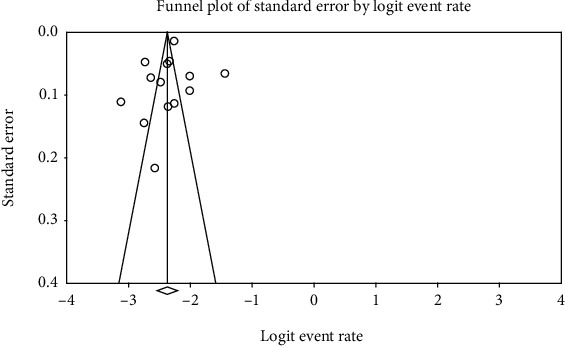
Funnel plot of results on the prevalence of low birth weight in Iranian infants.

Heterogeneity in articles was assessed by the *I*^2^ test, and based on this test, the value of 96% was achieved and is a representative of high heterogeneity in entered studies; therefore, the random effects model was used to combine the studies' results; obtained results from evaluation of publication bias in the studies were assessed by the Egger test (Figure 2) by which the bias was not statistically significant (*P* = 0.537).

**Figure 3:**
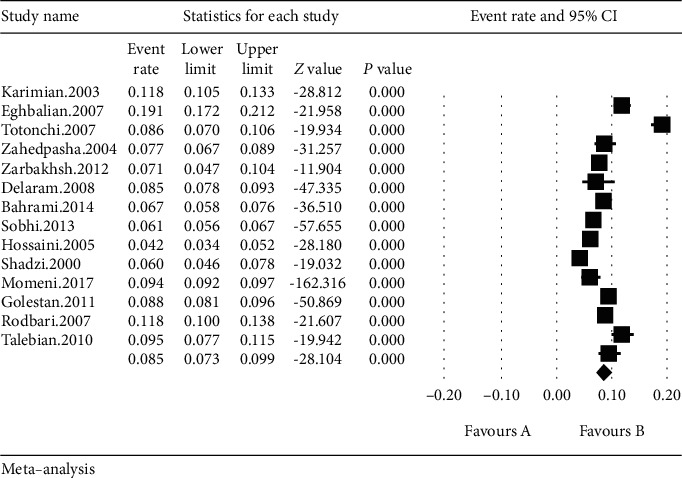
Total prevalence of LBW in Iranian infants based on the random effects model.

The total number of samples entered in the study was 93924 infants, and the overall prevalence of LBW in Iranian infants based on meta-analysis has been achieved at 8.5% (95% CI: 7.3-9.9%). The highest incidence of LBW was in infants in Hamadan at 19.1% (95% CI: 17.2-21.2%) [14] in 2007, and the lowest prevalence of LBW was in infants in Tonekabon at 4.2% (95% CI: 3.4-5.2%) [21] in 2005 (Figure 3). In this figure, the prevalence of LBW is shown based on the random effects model, by which the black square is the rate of prevalence, and the length of the segment by which the square lays on is the confidence interval for each study; the diamond sign shows the prevalence in the whole country for all the studies.

### 3.2. Metaregression Test

**Figure 4:**
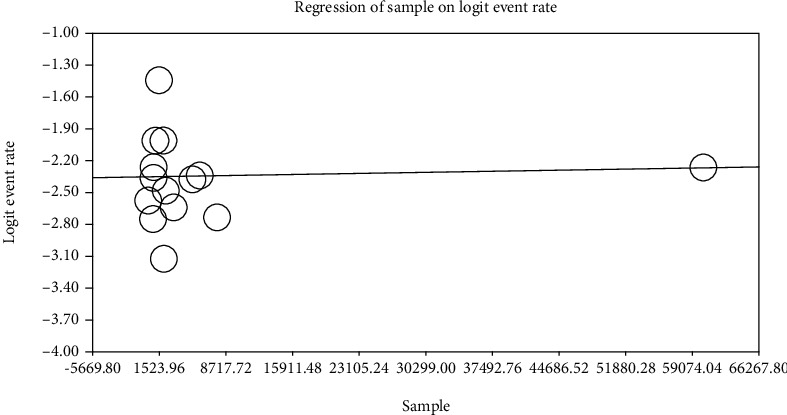
Metaregression chart on the prevalence of LBW in infants in Iran divided into sample size.

**Figure 5:**
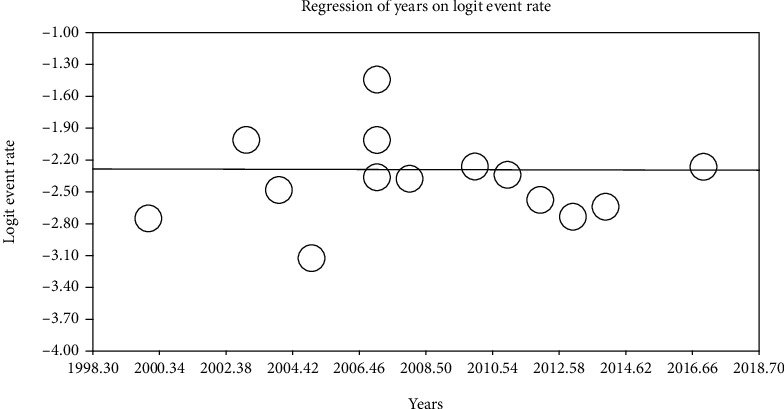
Metaregression chart on the prevalence of LBW in infants in Iran divided into year of investigation.

To investigate the impacts of active potential factors in the heterogeneity of prevalence of LBW in infants in Iran, a meta-regression test has been used on two factors of sample size and year of investigation (Figures 4 and 5). Based on Figure 4, by increasing the sample size, the total prevalence of LBW in infants in Iran also increases, by which this difference is statistically significant (*P* < 0.05).  Figure 5 also reported that by increasing the year of investigation, the total prevalence of LBW in infants in Iran also increases, by which this difference is not statistically significant (*P* = 0.820).

## 4. Discussion

Based on the results obtained from this study and investigations on 93924 infants, the total prevalence of LBW in infants in Iran according to the meta-analysis was achieved at 8.5%. The World Health Organization (WHO) reported incidence of LBW in sub-Saharan Africa at 15%, in the Middle East and northern Africa at 11%, in East Asian countries at 10%, in southern Asia at 33%, in Latin America at 9%, in developed countries at 6%, and developing countries and the whole world at 18% and 17%, respectively [27–30].

Other studies show that the prevalence of LBW inborn infants was 15% in Kenya, 8% in Ethiopia, 13.6% in Saudi Arabia, and 6% in the United States [31].

The studies showed that most of the LBW infants are with intrauterine growth retardation, which can be due to nutritional deficiency, low maternal age, and lack of pregnancy care [32, 33], and other researches show that small maternal age (less than 18 years) can contribute to the birth of LBW infant [34].

These studies report that factors such as trauma to the mother's abdomen leading to preterm labor, membrane rupture, placental abruption or detachment, and uterine detachment and also factors such as nicotine and tobacco and alcohol use, low economic status of the family, low maternal weight, anemia, inappropriate diet, sexually transmitted diseases, and mental diseases can be of most important factors leading to LBW in infants [33–35].

The main symptom of IUGR is a small size for gestational age baby. Specifically, the baby's estimated weight is below the 10th percentile or less than that of 90% of babies of the same gestational age. Depending on the cause of IUGR, the baby may be small all over or look malnourished. They may be weak and pale and have loose, dry skin. The umbilical cord is often thin and dull instead of thick and shiny [33–35].

LBW's importance is due to the birth of girls with LBW, by which they are going to be mothers with a higher probability of giving birth to infants with LBW. In other words, delivery of LBW infants has transmitted intergeneration [36–39].

This condition in various conducted studies in low economic status or areas showed a higher prevalence of LBW so that the incidence of LBW was 39.1% in India's slum area, 36.8% in impoverished regions in Bangladesh, and 20.9% in rural areas of most Asian countries [36–41].

Due to the relatively high prevalence of LBW in Iran, it is necessary to increase the maternal health rate through care processes before and after labor, appropriate nutrition, and prevention of iron deficiency anemia and also through an increase in awareness of parents and mothers by mass media and developing care programs for mothers during pregnancy, and obligation on referral to health centers for mothers and implementing pregnancy care provide the basis for a decline in LBW in infants in-country, and also by equipping and promoting quality of intensive care units in the country, the fundamental basis for hospitalization and treatment of premature and LBW infants would be provided.

### 4.1. Limitation

The most important limitations of the current study are the lack of access to the full texts of articles and the low quality of some articles.

## 5. Conclusions

Given the high prevalence of LBW in infants in Iran, health policy-makers must produce effective policies to take sufficient management attempts, including periodical care for pregnant mothers and infants.

## Figures and Tables

**Table 1:** Specifications of studies entered in the study.

Row	Author (references)	Publication year	Area	Sample size	Prevalence
1	Karimian et al. [13]	2003	Qom	1972	11.8
2	Eghbalian [14]	2007	Hamedan	1500	19.1
3	Tootoonchi [15]	2007	Tehran	905	8.6
4	Zahed Pasha et al. [16]	2004	Babol	2228	7.7
5	Zarbakhsh et al. [17]	2012	Gilan	325	6.9
6	Delaram and Ahmadi [18]	2008	Shahr-e-kord	5102	8.5
7	Bahrami et al. [19]	2014	Qazvin	3076	6.7
8	Sobhi et al. [20]	2013	Mashhad	7763	6.1
9	Hosseini et al. [21]	2005	Tonekabon	2016	4.2
10	Shadzie et al. [22]	2000	Isfahan	848	6
11	Momeni et al. [23]	2017	Kerman	60273	9.4
12	Golestan et al. [24]	2011	Yazd	5897	8.8
13	Roudbari et al. [25]	2007	Zahedan	1109	11.8
14	Talebian and Afrouz [26]	2010	Isfahan	910	9.5

## Data Availability

Datasets are available through the corresponding author upon reasonable request.

## Abbreviations

WHO: World Health Organization
LBW: Low birth weight
SID: Scientific Information Database
STROBE: STrengthening the Reporting of OBservational studies in Epidemiology for cross-sectional study
PRISMA: Preferred Reporting Items for Systematic Reviews and Meta-Analyses.
